# Evaluation of the putative lymphoma-associated point mutation D427H in the STAT3 transcription factor

**DOI:** 10.1186/s12860-022-00422-9

**Published:** 2022-06-25

**Authors:** Lena Sophie Behrendsen, Priyanka Rajeev Menon, Muhammad Jawad Khan, Anke Gregus, Oliver Wirths, Thomas Meyer, Julia Staab

**Affiliations:** 1 Department of Psychosomatic Medicine and Psychotherapy, University Medical Centre Göttingen, 37073 Göttingen, Germany; 2 German Centre for Cardiovascular Research (DZHK), Partner Site Göttingen, Göttingen, Germany; 3 Department of Biosciences, COMSATS University Islamabad, 45550, Islamabad, Pakistan; 4 Department of Psychiatry and Psychotherapy, University Medical Centre, Göttingen, Germany

**Keywords:** Lymphoma-associated missense mutation, Signal transducer and activator of transcription 3 (STAT3), Interleukin-6 signaling, DNA binding, Gene expression

## Abstract

**Background:**

Signal transducer and activator of transcription 3 (STAT3) is an oncogenic transcription factor that promotes cell proliferation and immunomodulation in untransformed cells and maintains stemness of transformed cells, facilitating invasion and metastasis. Numerous point mutations in the STAT3 protein have been identified that drive malignancy in various tumor entities. The missense mutation D427H localized in the STAT3 DNA-binding domain has been previously reported in patients with NK/T cell lymphomas. To assess the biological activity of this missense mutation, we compared the STAT3-D427H mutant to wild-type (WT) protein as well as the known hyper-active mutant F174A.

**Results:**

Although previously reported as an activating mutation, the STAT3-D427H mutant neither showed elevated cytokine-induced tyrosine phosphorylation nor altered nuclear accumulation, as compared to the WT protein. However, the D427H mutant displayed enhanced binding to STAT-specific DNA-binding sites but a reduced sequence specificity and dissociation rate from DNA, which was demonstrated by electrophoretic mobility shift assays. This observation is consistent with the phenotype of the homologous E421K mutation in the STAT1 protein, which also displayed enhanced binding to DNA but lacked a corresponding increase in transcriptional activity.

**Conclusions:**

Based on our data, it is unlikely that the D427H missense mutation in the STAT3 protein possesses an oncogenic potential beyond the WT molecule.

**Supplementary Information:**

The online version contains supplementary material available at 10.1186/s12860-022-00422-9.

## Background

Signal transducers and activators of transcription (STATs) are a group of cytokine-driven transcription factors that initiate and modulate numerous proliferative and immunological processes inside the cell [1]. The highly conserved STAT signaling pathway is used by a myriad of cytokines and growth factors to influence cellular function. In response to cytokine stimulation, STATs are activated by cytokine receptor-associated Janus kinases (JAKs) that phosphorylate a critical tyrosine residue in the STAT transactivation domain. This facilitates dimerization and subsequent translocation to the nucleus to initiate the transcription of cytokine-responsive genes. All STATs share a common structural organization comprising of 6 domains: 1) an amino-terminal domain, 2) a coiled-coil domain, 3) a DNA-binding domain, 4) a linker domain, 5) an Src-homology 2 (SH2) domain, and (6) a C-terminal transactivation domain [2, 3]. In the human STAT family, STAT3 was initially identified as acute phase response factor (APRF) that is rapidly activated by interleukin-6 (IL-6) in hepatic inflammation. Intensive investigation revealed that APRF is additionally activated in response to interferon-γ (IFNγ) and shares homology with other discovered STAT family members [4]. The IL-6 cytokine family functions through receptors that are heterodimers comprising membrane glycoprotein 130 kDa (gp130, also known as IL-6Rβ). The gp130 receptor associates with JAKs on their intracellular side which phosphorylate the tyrosine residue 705 in the transactivation domain of STAT3 [5–7]. STAT3 monomers can dimerize in two possible orientations: either a parallel dimer through reciprocal interactions between the phosphotyrosine and the SH2 domain or an anti-parallel dimer through inter-molecular interactions between the coiled-coil domain and DNA-binding domain [8–11].

Through activation by IL-6 and other cytokines, STAT3 has been implicated in many immunological processes, including the induction of hepatic acute phase proteins, stimulation of cell proliferation and differentiation in lymphocytes, maintenance of pluripotency in embryonic stem cells, and inhibition of apoptosis [1, 12]. Oncogenic mutations in the STAT3 protein have been identified, wherein its hyper-phosphorylation, premature nuclear retention, enhanced DNA-binding affinity, and subsequent upregulation of target genes are known to drive several hallmarks of tumorigenesis, including metastasis, immune evasion, and drug resistance. Due to its regulatory role in cell proliferation and differentiation, mutations in STAT3 have been associated with poor prognosis in several cancers. Several activating mutations in the SH2 domain of STAT3, such as D661V and D661Y, have been identified in T-cell large granular lymphocytic leukemia (T-LGL) patients [13]. Mutation V637M has been reported to drive hyper-IgE syndrome, while the Y640F mutation has been linked to the development of several malignancies [14]. The DNA-binding domain and coiled-coil domain of STAT3 have also been reported to harbour mutations that cause auto-immunity and lymphoproliferation [15].

In patients with mature T-cell lymphomas and their subtypes, peripheral T-cell lymphoma (PTCL) and NK/T-cell lymphomas (NKTL), *STAT3* is among the most frequently mutated genes [16]. Four novel STAT3 mutations (D427H, E616G, E616K and E696K) were previously discovered in patients with mature T-cell lymphomas and reported to be damaging and gain-of-function (GOF) using FATHMM (functional analysis through hidden Markov models) assessment. These mutants were described as constitutively hyper-phosphorylated and increased the transcription of STAT3 target genes compared to the wild-type (WT) molecule [16]. Since the D427H substitution in STAT3 is homologous to E421K mutation in STAT1, we chose to study the molecular mechanism underlying the hyper-activity of the disease-associated D427H mutant. The E421K mutant of STAT1 displayed an enhanced gamma-activated site (GAS) binding, a low off-rate from DNA and significantly reduced gene activation [17]. Therefore, we were intrigued by the supposed increase in the oncogenic potential of the D427H mutation in STAT3 and replaced the Asp^427^ in the DNA-binding domain with histidine (D427H) through site-directed mutagenesis. The phenotype of this mutant was then compared to the WT protein and to the previously characterized, activating GOF mutation F174A in the STAT3 coiled-coil domain, which was used as a positive control [18].

## Material and methods

### Plasmids, mutagenesis and cell culture

This study was performed using two expression plasmids, namely pSTAT3-GFP encoding a carboxy-terminal fusion protein of the full-length murine STAT3 cDNA with green fluorescent protein (GFP) and pSTAT3-SNAP with a SNAP tag [10, 18]. The two vectors were received as a kind gift from Prof. Gerhard Müller-Newen from the Universitätsklinikum RWTH Aachen, Germany. The QuikChange II kit from Stratagene was used to perform site-directed mutagenesis to introduce point mutations in the expression plasmids. For this purpose, we used the following primers with mutated codons underlined (only forward primers are shown):

D427HF; 5´- GGAGGCCGTGCCAATTGTCATGCCTCCTTGATCGTGACTG -´3,

F174AF; 5´- CTCCAGGACGACTTTGATGCCAACTACAAAACCCTCAAG -´3,

All point mutations were confirmed by standard dideoxy-termination DNA sequencing (Microsynth Seqlab, Göttingen) and the generated vectors were used for transfection experiments. In addition to HeLa-S3 cells, STAT1-negative U3A cells were transfected with the corresponding plasmids in order to control for the interference of co-expressed STAT1 on STAT3 signal transduction [19]. These cell lines were a kind gift from Prof. Uwe Vinkemeier, University of Nottingham, United Kingdom. The cells were cultured in a humidified 5% CO_2_ atmosphere at 37 °C in Dulbecco’s modified Eagle’s medium (DMEM) (for U3A cells; PAA Laboratories) or Roswell Park Memorial Institute 1640 medium (RPMI) (for HeLa cells; Lonza) supplemented with 10% fetal calf serum (FCS; Biochrom), 100 IU/ml penicillin, 100 IU/ml streptomycin, and 0.04 μg/ml puromycin (only for U3A cells; Sigma-Aldrich). Cells were transfected with MegaTran2.0 (Origene) and on the next day stimulated with 25 ng/ml of recombinant human IL-6 (Gibco) or 50 ng/ml of recombinant human IFNγ (Biomol) for the indicated times. For EMSA extracts, STAT3-variant expressing cells were stimulated for 30 min with the cytokines indicated.

### Protein extraction and detection

Cells expressing recombinant STAT3 tagged with either GFP- or SNAP were grown on 6-well dishes and lysed on ice for 5 min in 50 µl cytoplasmic extraction buffer (20 mM HEPES, pH 7.4, 10 mM KCl, 10% (v/v) glycerol, 1 mM EDTA, 0.1 mM Na_3_VO_4_, 3 mM 1,4-dithiothreitol [DTT], 0.1% IGEPAL-CA-360, 0.4 mM Pefabloc [Sigma-Aldrich], and Complete Mini protease inhibitors [Roche]). The lysates were centrifuged for 15 s at a temperature of 4 °C and 16,000 g. The supernatants were spun again for 5 min and collected as cytoplasmic extracts. The pellets reserved from the first centrifugation step were resuspended in a volume of 50 µl nuclear extraction buffer (20 mM Hepes, pH 7.4, 420 mM KCl, 20% (v/v) glycerol, 1 mM EDTA, 3 mM DTT, 0.1 mM Na_3_VO_4_, 0.4 mM Pefabloc, and Complete Mini protease inhibitors) and incubated for 30 min on ice. Subsequently, these samples were centrifuged for 15 min and 4 °C at 16,000 g and collected as nuclear extracts. Equal amounts of nuclear and the corresponding cytoplasmic extracts were mixed for each sample. The combined extracts were then boiled for 3 min in sodium dodecyl sulphate (SDS) sample buffer and resolved by 10% SDS–polyacrylamide gel electrophoresis (PAGE) with subsequent transfer onto poly-vinylidene difluoride (PVDF) membranes. The membranes were then blocked with 25% bovine serum albumin in Tris-buffered saline with 0.05% Tween-20. These blots were then incubated with either the monoclonal phospho-Tyr705-specific STAT3 antibody D3A7 or the monoclonal pan-STAT3 antibody D1B2J, both obtained from Cell Signaling, and after three washing steps exposed to the conjugated secondary anti-rabbit IRDye 800CW antibody (LI-COR). Bound immunoreactivity on the membrane was detected using the LI-COR Odyssey imaging system. The pSTAT3 band intensity was normalized to the corresponding amount of total STAT3 for each variant and stimulation condition.

### Fluorescence microscopy

Direct fluorescence microscopy was employed to monitor the kinetics of IL-6- and IFNγ-induced nuclear accumulation of STAT3 variants in U3A and HeLa cells [18]. Cells expressing GFP- or SNAP-tagged STAT3 grown in 8-well chamber slides were stimulated with cytokines, as indicated. SNAP-tagged STAT3 was visualized by staining the cells for 30 min with the SNAP-tag substrate SNAP-Cell TMR-Star from New England Biolabs. Thereafter, cells were washed thrice with pre-warmed culture media and rested for 30 min in their media before being stimulated with cytokines. At the end of stimulation, cells were fixed for 15 min with 4% paraformaldehyde in phosphate-buffered saline (PBS) at room temperature and subsequently nuclei were stained with 5 μg/ml of Hoechst dye 33258 (Sigma-Aldrich) for 10 min. For antibody staining, cells expressing GFP- or SNAP-tagged STAT3 were, after cytokine stimulation, fixed with methanol for 15 min at -20 °C and subsequently permeabilized with 1% Triton X-100/PBS at room temperature (RT) for 20 min. To saturate unspecific binding sites, the cells were treated with 25% FCS in PBS for 45 min while being shaken. This was followed by a 45-min shaking at RT with the primary antibody (monoclonal rabbit phospho-Tyr705-specific STAT3 antibody, Cell Signaling, D3A7; 1:1000 in 25% FCS/PBS). The cells were then washed thrice with PBS. For the detection of the primary phospho-STAT3 antibody, a Cy3-coupled anti-rabbit IgG secondary antibody from goat (Jackson Immunoresearch Laboratories, USA; 1:1000 in 25% FCS/PBS) was added and incubated for 45 min at RT while being shaken, followed by nuclear staining. Slides were mounted in a fluorescence mounting medium, which was obtained from Southern Biotech. Intracellular fluorescence staining patterns were visualized using a Nikon Eclipse Ti fluorescence microscope, which was equipped with appropriate filters. Images taken with a Nikon DS-Qi2 camera were further processed with the NIS elements software (Nikon). Nuclear and total cellular fluorescence intensities were determined using ImageJ (NIH) and normalized to the background intensity of the respective images. Mean nuclear-to-total cellular fluorescence intensity ratios including their standard deviations were calculated from 20 randomly selected transfected cells, for each variant.

### Electrophoretic mobility shift assay

STAT3 proteins were examined by means of electrophoretic mobility shift assays (EMSA) for their binding to specific or mutant duplex oligonucleotides containing a single sis-inducible element (SIE)/GAS element. Cellular extracts (4 µl) from IL-6- or IFNγ-stimulated cells expressing recombinant STAT3 were incubated with EMSA reaction buffer (8 µl) containing the unspecific, synthetic competitor poly(deoxyinosinic-deoxycytidylic) acid and 1 ng of the [^33^P]-labelled duplex oligonucleotide probe, which was generated by an end-filling reaction using the Klenow fragment (New England Biolabs). A duplex oligonucleotide M67 with a single, canonical SIE/GAS site was used to test the binding of STAT3 variants (SIE/GAS site is underlined, anti-sense oligo is not shown): 5´-TTTTCGACATTTCCCGTAAATCTG-´3. To test for changes in sequence-specific DNA binding of the STAT3 mutants, additional oligonucleotides with two complete GAS sites in tandem orientation (2xGAS) or one complete GAS site (GAS-nonGAS) or no GAS site (2xnon-GAS) were used:

2xGAS; 5′-TTTTCGTTTCCCCGAAATTGACGGATTTCCCCGAAAC-′3,

GAS-nonGAS; 5′-TTTTCGTTTCCCCGAAATTGACGGATTTACCCCAAC-′3,

2xnonGAS; 5′-TTTTCGTTTACCCCAAATTGACGGATTTACCCCAAC-′3.

For competition experiments, cellular extracts were incubated with [^33^P]-labelled duplex M67 oligonucleotides for 15 min at RT in EMSA buffer. Subsequently, a 750-fold molar excess of unlabelled M67 DNA was added for 10 min on ice. The reactions were loaded on a 4.8% acrylamide:bisacrylamide (29:1) gel at 4 °C and separated at 400 V. Sequence-specific DNA binding was visualized on vacuum-dried gels using the laser phosphorimaging system Typhoon FLA 9500 (GE Healthcare Life Sciences). The band intensity corresponding to the STAT3 variants on the autoradiograms was either measured as an absolute value or divided by the intensity of a fast-migrating unspecific band, which was regarded as a loading control and is labelled with an asterisk in the figures.

### Reporter gene assays and real-time PCR

Promoter activation mediated by STAT3 was studied in transfected U3A cells using a vector encoding a luciferase reporter with three copies of an IFNγ-inducible Ly6E GAS element in the promoter region upstream of the transcriptional start site [20, 21], as described in our previous paper [18]. U3A cells grown on 48-well plates were co-transfected with three vectors in each well: the luciferase reporter 3xLy6E (70 ng), a β-galactosidase plasmid (200 ng), and an expression plasmid encoding for either GFP- or SNAP-tagged WT or mutant STAT3 (250 ng). Twenty-four hours after transfection, cells were either untreated or treated with IL-6 or IFNγ for 6 h, before cellular extracts were prepared using a lysis buffer containing 25 mM glycylglycine, 1% Triton X-100, 15 mM MgSO_4_, 4 mM EGTA, 0.4 mM Pefabloc, 3 mM DTT, pH 7.8, and Complete protease inhibitors. Reporter gene expression was assessed by means of a luciferase assay system from Promega using the luminometer Centro KS LB960 (Berthold Technologies). Luciferase expression was first normalized to the β-galactosidase activity, which was determined spectroscopically at a wavelength of 420 nm in the corresponding samples. Five independent transfections were tested for each STAT3 variant and stimulation mode, and the experiment was repeated in duplicate.

To test endogenous target gene induction, U3A cells expressing STAT3-GFP were cultured for 18 h in Dulbecco’s modified Eagle’s medium supplemented with 1% FCS, prior to stimulation for 3 h with IL-6 or IFNγ. RNA was isolated from the cells using the peqGold Total RNA kit (VWR Lifesciences). First-strand cDNA synthesis was carried out using the Verso cDNA Synthesis kit (Thermo Fisher Scientific). Real-time PCR reactions were performed in a volume of 20 µl, containing 25 ng cDNA, 70 nM of each specific primer, and 10 µl of Absolute Blue qPCR SYBR Green Mix (Thermo Fisher Scientific). The following primer pairs were used, according to sequence data available from the National Center for Biotechnology Information (NCBI):

*hCyclinD1*F; 5'-CGG TGT CCT ACT TCA AAT GT-3',

*hCyclinD1*R; 5'-ATG GAG TTG TCG GTG TAG AT-3',

*hc-Myc*F; 5´-GGTCTTCCCCTACCCTCTCAACGA-´3,

*hc-Myc*R; 5´-GGCAGCAGGATAGTCCTTCCGAGT-´3,

*hGAPDH*F; 5´-GAAGGTGAAGGTCGGAGTC-´3, and.

*hGAPDH*R; 5´-GAAGATGGTGATGGGATTTC-´3.

The PCR protocol included a denaturation step at 95 °C for 15 min and 40 cycles of denaturation at 95 °C for 15 s, annealing at 55 °C for 30 s, and extension at 72 °C for 30 s. After the final amplification step, a melting curve analysis was performed on the Eppendorf Mastercycler ep realplex 2 using a temperature gradient from 60 °C to 95 °C in 0.5 °C increment steps and fluorescence being measured at each temperature for a period of 10 s. The relative expression of a target transcript was normalized to the expression of the *GAPDH* gene. The △△ Ct-method was used to determine comparative relative expression levels, based on the formula 2^−(△Ct target − △Ct reference sample)^. All real-time PCR reactions were performed in two independent transfection experiments with duplicate reactions each.

### Data analysis

ImageJ (NIH) software was used to process digital images and data figures were created using CorelDRAW Graphics Suite 2019. Means and standard deviations were calculated for each STAT3 variant and stimulation condition. Data were analysed using the program GraphPad PRISM. Differences between the groups were assessed using Student’s *t*-test, and a *p*-value ≤ 0.05 was considered to indicate statistical significance.

## Results

### The residue D427 is located in a flexible region of the STAT3 DNA-binding domain that is spatially close to DNA

**Fig. 1:**
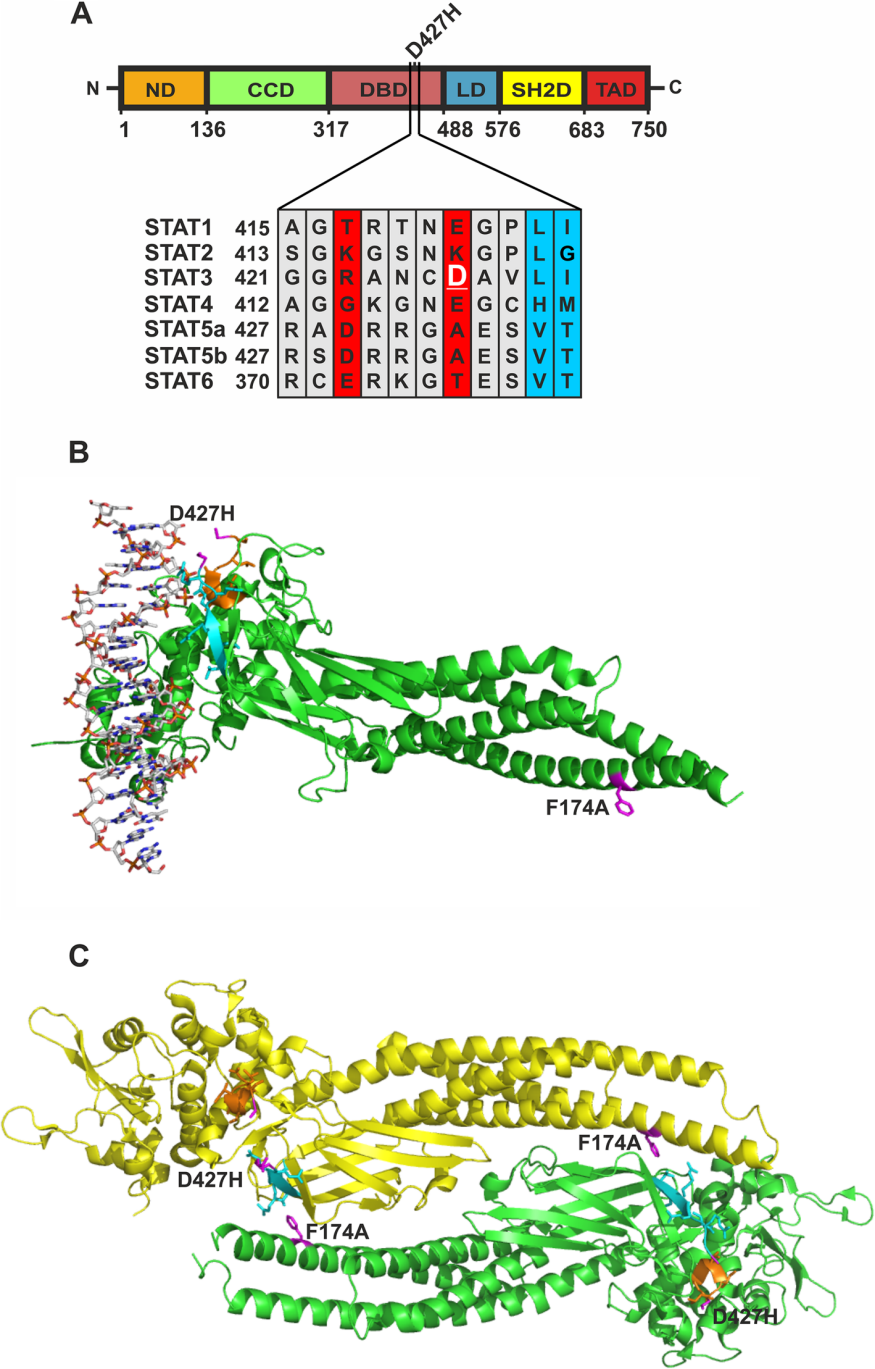
**A**–**C**: The D427 residue is localized in the DNA-binding domain of STAT3 but its atomic coordinates cannot be precisely defined due to the steric flexibility of this region. **A** Multiple sequence alignments of the regions surrounding D427 residue across the seven STAT family members show STAT1, STAT3, STAT4, and STAT6 to house polar amino acids at homologous positions. **B** Crystal structure of STAT3 bound to DNA highlighting the region surrounding the mutation D427H and the location of the crucial point mutation F174A (only one protomer is shown). The surrounding region to D427 towards to the amino-terminus is marked in cyan while the region towards the carboxy-terminus is highlighted in orange. Adjacent residues to D427 on either side as well as the F174 residue in the coiled-coil domain are highlighted in magenta. **C** Crystal structure of an anti-parallel dimer of STAT3 showing the same residues and regions as described above. Structural data were obtained from the Protein Data Bank (pdb) file 4E68 for the parallel STAT3 dimer [8] and 6TLC [11] for the STAT3 anti-parallel dimer, and modified using PyMOL (De Lano Scientific)

In the common STAT modular structure, the DNA-binding domain is quite conserved across all STAT proteins. Sequence alignment of STAT family members shows that a negatively charged amino acid residue is located at position 427 in STAT3 (Fig. 1A). Within the crystal structure of a DNA-bound STAT3 dimer the position of the D427 residue was not resolved, as its atomic coordinates could not be precisely defined due to the structural flexibility of this region. In Fig. 1B and C, the adjacent sequences of the residue D427 are marked in cyan (towards N-terminus) and orange (towards C-terminus). It is evident from Fig. 1B that the sequence region of the point mutation D427H itself must be in close spatial relationship to the DNA, despite the steric flexibility of this region. Due to the large spatial distance to DNA (Fig. 1B, C), the mutation F174A in the coiled-coil domain has no influence on DNA-binding but the F174 residue is essentially involved in the formation of the antiparallel conformation of the STAT3 dimer (Fig. 1C).

### STAT3-D427H displays unchanged phosphorylation kinetics compared to the WT protein

**Fig. 2:**
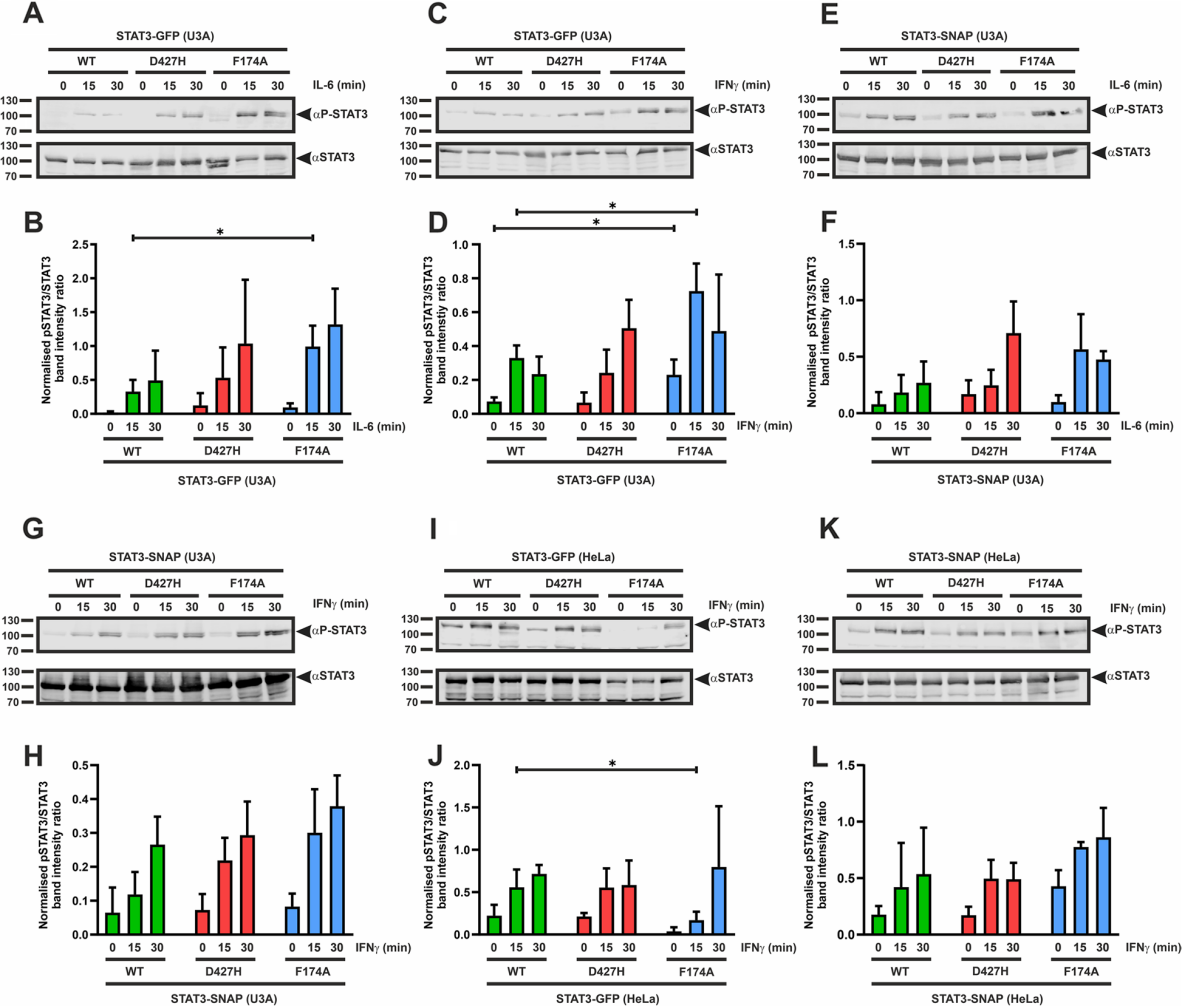
**A**–**L**: D427H mutation does not alter tyrosine phosphorylation of STAT3 upon cytokine stimulation. U3A and HeLa cells were transfected with GFP/SNAP-tagged variants of STAT3 and subsequently stimulated with different cytokines for the indicated times. **A** Representative immunoblot of whole cell extracts from U3A cells expressing GFP-tagged STAT3 proteins after treatment for 0, 15, and 30 min with 25 ng/ml of recombinant IL-6 and the quantification thereof from three independent transfection experiments (**B**). Similar experiments were performed with GFP-tagged STAT3 variants after 50 ng/ml IFNγ stimulation as shown in the representative immunoblot (**C**) and the quantification thereof from three independent transfection experiments (**D**). **E** Western blot of whole cell extracts from U3A cells expressing SNAP-tagged STAT3 variants after treatment for 0, 15, and 30 min with recombinant IL-6 and the quantification thereof from three independent transfection experiments (**F**). **G** Representative immunoblot of whole cell extracts from U3A cells expressing SNAP-tagged STAT3 mutants after treatment for 0, 15, and 30 min with recombinant IFNγ and the quantification thereof from three independent transfection experiments (**H**). **I**–**L** Western-blotting experiments demonstrating unchanged kinetics of tyrosine phosphorylation of STAT3-D427H as compared to WT and the positive control F174A performed in HeLa cells expressing the STAT3 variants which were subsequently stimulated with 50 ng/ml of IFNγ for the indicated times. **I** Representative immunoblot of whole cell extracts from HeLa cells expressing GFP-tagged STAT3 mutants after IFNγ stimulation and the quantification thereof from three independent transfection experiments (**J**). Similar experiments were performed with SNAP-tagged STAT3 variants as shown in a representative immunoblot (**K**) and the quantification thereof from three independent transfection experiments (**L**). Means ± standard deviations from three independent experiments with *p ≤ 0.05. Arrows indicate the respective STAT3-GFP or STAT3-SNAP bands of interest

To investigate changes in tyrosine phosphorylation of STAT3 upon mutation of the D427 residue in the DNA-binding domain, we performed Western-blotting experiments using lysates from IL-6- or IFNγ-stimulated, STAT1-deficient U3A cells expressing the STAT3 variants (Fig. 2). We transiently transfected U3A cells with plasmids coding for GFP-tagged variants of STAT3 and on the following day stimulated them with 25 ng/ml of IL-6 or 50 ng/ml of IFNγ for 0, 15 and 30 min, prior to total protein extraction. As seen in Fig. 2A–D, STAT3-D427H showed a level of tyrosine phosphorylation that was similar to the WT protein. We also examined the time-dependent tyrosine phosphorylation of SNAP-tagged STAT3-D427H expressed in U3A cells, which was not significantly altered from the phosphorylation kinetics of the WT (Fig. 2E–H). Furthermore, the unchanged phosphorylation of the D427H mutant was also seen in lysates from IFNγ-stimulated HeLa cells (Fig. 2I–L). In contrast, the F174A mutant showed hyper-phosphorylation compared to the WT and D427H proteins, due to its failure to stabilize the anti-parallel dimer conformation which is imperative for STAT3 dephosphorylation [18].

### STAT3-D427H shows similar kinetics of nuclear accumulation as the WT protein

Premature or prolonged nuclear accumulation of STAT3 could attribute to its hyper-activity and oncogenic potential. Therefore, we tested the impact of the D427H mutation on the nucleo-cytoplasmic distribution of STAT3 through direct and indirect immunofluorescence experiments. U3A cells were transfected with plasmids expressing GFP- and SNAP-tagged variants of STAT3 and on the next day stimulated with either IL-6 or IFNγ for the indicated time durations. Post-stimulation, cells expressing the SNAP-tagged constructs were stained with a fluorescently labelled substrate for enzymatic cleavage by the SNAP tag and subsequent visualization. Thereafter, cells expressing GFP- and SNAP-tagged STAT3 variants were subjected to an additional indirect immunofluorescence staining by means of an antibody against phosphotyrosine 705 of STAT3.

**Fig. 3:**
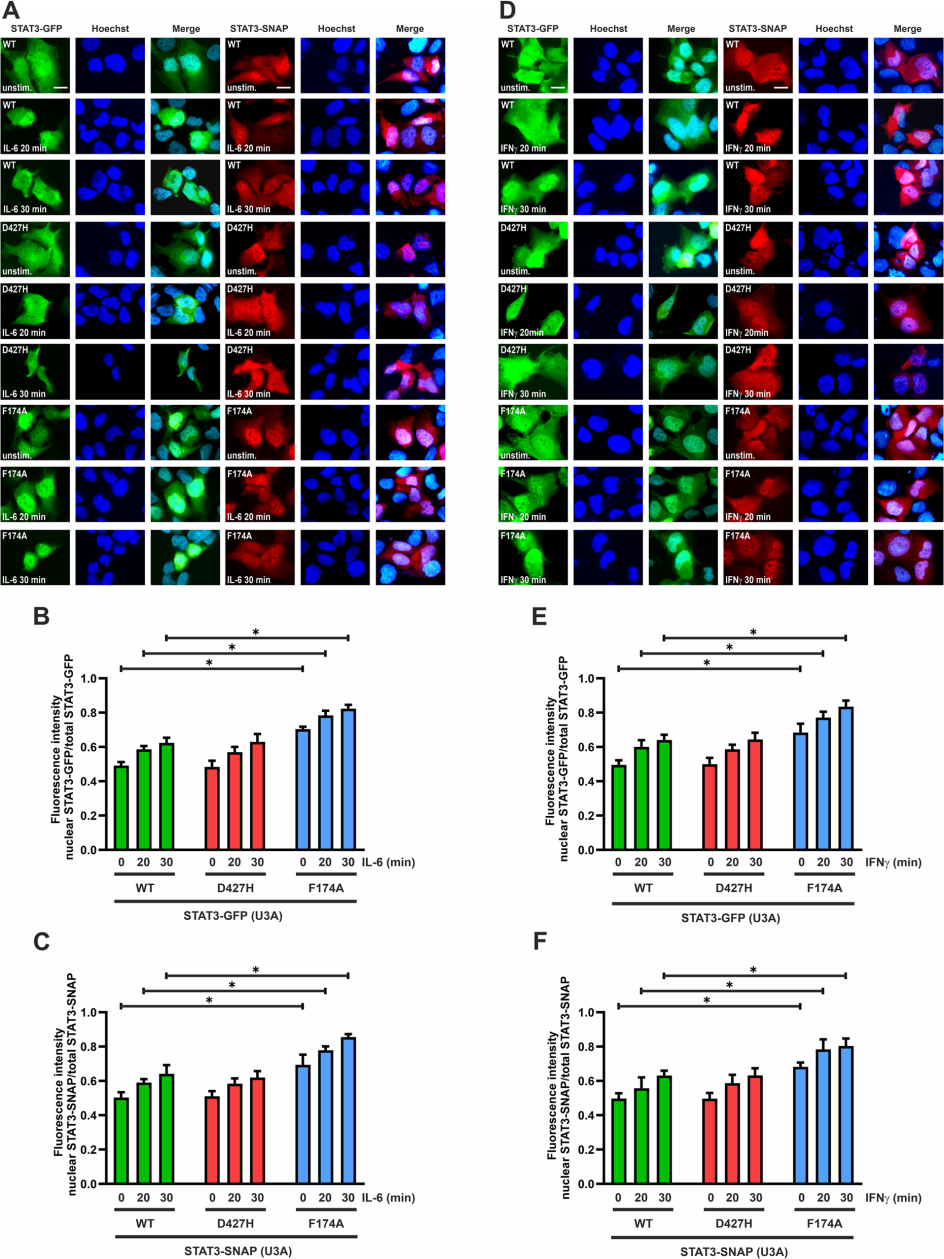
**A**–**F**: The D427H mutation does not alter cytokine-induced nuclear translocation of STAT3. U3A cells were transfected with GFP- and SNAP-tagged STAT3 variants and stimulated with either 25 ng/ml of IL-6 or 50 ng/ml of IFNγ for the indicated times. **A** The fluorescence micrographs show the intracellular distribution of GFP/SNAP-tagged WT and mutant STAT3, as well as the localization of the corresponding Hoechst-stained nuclei in cells stimulated with IL-6 for 0, 20 and 30 min. Histograms demonstrating the unaltered kinetics of D427H-GFP (**B**) and D427H-SNAP (**C**) nuclear accumulation as compared to the WT upon stimulation with IL-6, as determined by the ratio of nuclear-to-total fluorescence intensity (*n* = 3 independent transfections). **D** The fluorescence micrographs show the intracellular distribution of GFP/SNAP-tagged WT and mutant STAT3, as well as the localization of the corresponding Hoechst-stained nuclei following IFNγ stimulation. Histograms demonstrating the unchanged kinetics of D427H-GFP (**E**) and D427H-SNAP (**F**) nuclear accumulation compared to the WT protein, as determined by the ratio of nuclear-to-total fluorescence intensity (*n* = 3 independent transfections). Means ± standard deviations from *n* = 20 cells with *p ≤ 0.05

Fluorescence micrographs in Fig. 3 showed that prior to cytokine stimulation both GFP- and SNAP-tagged STAT3-WT were predominantly cytoplasmic, and gradually accumulated in the nucleus with increasing duration of cytokine exposure. While there was no observed difference in the resting distribution between WT and the D427H mutant, the positive control STAT3-F174A already showed a prominent nuclear presence before stimulation. However, despite of reportedly having enhanced oncogenic potential, the GFP-tagged STAT3-D427H did not differ from WT-GFP in the kinetics of nuclear accumulation upon IL-6 stimulation (Fig. 3A, B). Following IL-6 exposure, SNAP-tagged STAT3-D427H also accumulated in a similar fashion as the WT-SNAP protein (Fig. 3A, C).

**Fig. 4:**
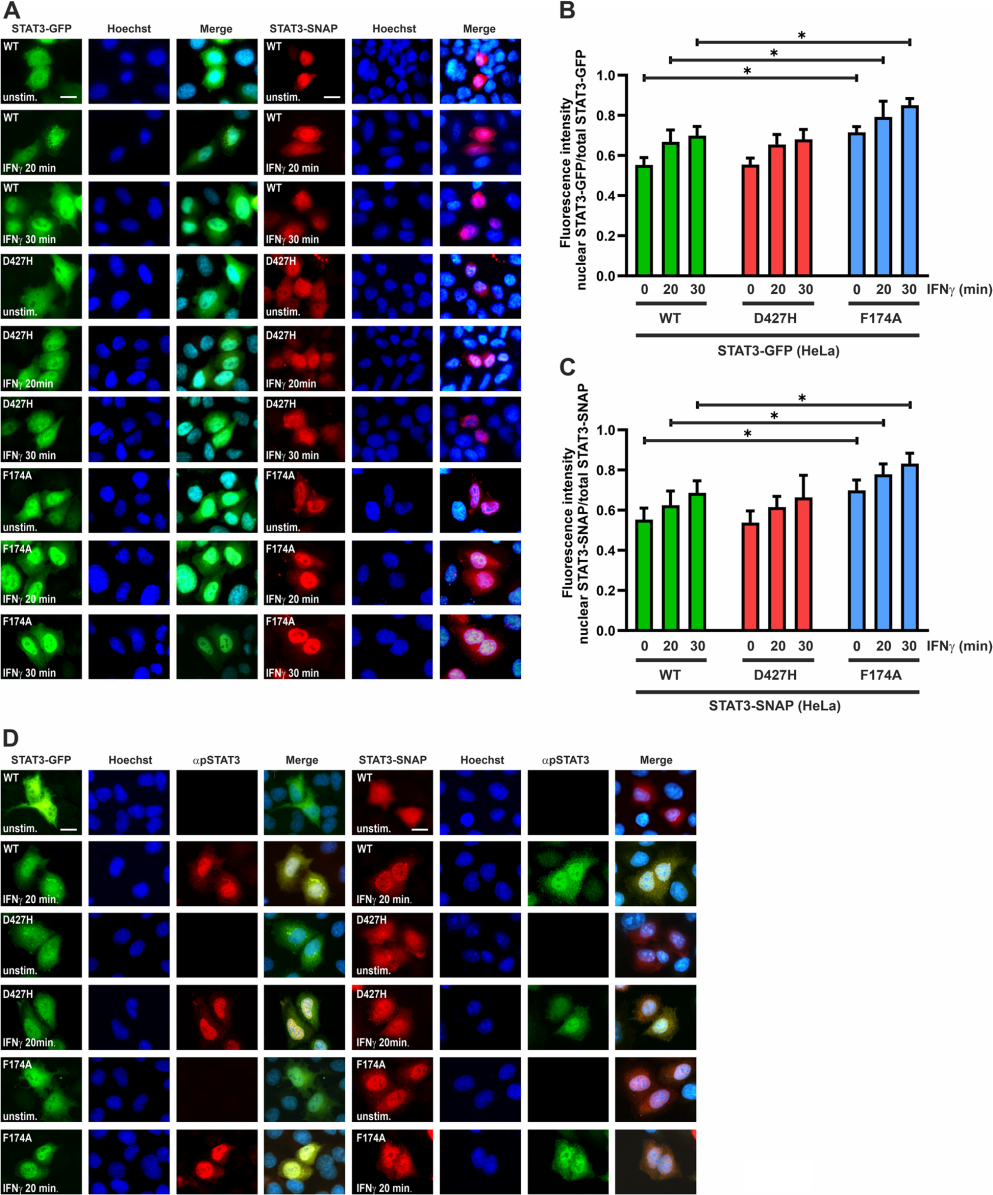
**A**–**D**: Unchanged nucleo-cytoplasmic translocation. **A–C** HeLa cells were transfected with expression plasmids coding for either GFP- or SNAP-tagged mutants of STAT3 and stimulated for indicated times with 50 ng/ml of recombinant IFNγ. Fluorescence micrographs show unaltered nuclear accumulation of D427H-GFP and D427H-SNAP as compared to the hyper-phosphorylated F174A mutant, which served as a positive control. Histograms show the quantification of nuclear STAT3-GFP/-SNAP as a ratio of total cellular GFP/SNAP intensity from three independent experiments (*n* = 3). **D** Indirect immunofluorescence using an antibody against the phospho-tyrosine 705 residue in STAT3 confirms the translocation of phospho-STAT3 in response to IFNγ stimulation in transfected HeLa cells. GFP- and SNAP-tagged STAT3 variants were transfected in HeLa cells which were subjected to stimulation with 50 ng/ml recombinant IFNγ for the indicated times. These cells were fixed and stained with an antibody against phospho-STAT3, followed by a secondary antibody (Cy3 (red) for cells expressing STAT3-GFP and Cy2 (green) for cells expressing STAT3-SNAP). Means ± standard deviations from *n* = 20 cells with *p ≤ 0.05

We also tested the nuclear accumulation of the STAT3 variants upon stimulation with IFNγ. Both SNAP- and GFP-tagged D427H mutants displayed unaltered nuclear accumulation as compared to their corresponding WT fusion proteins (Fig. 3D–F). Irrespective of the cytokine used, both GFP- and SNAP-tagged F174A mutant showed an enhanced nuclear accumulation as compared to the WT. We also tested the kinetics of STAT3-D427H nuclear accumulation in HeLa cells expressing either the GFP- or SNAP-tagged variant after stimulation with IFNγ. The D427H mutant showed unchanged kinetics of cytokine-induced nuclear accumulation as compared to the WT protein, while the positive control F174A showed a premature nuclear presence and enhanced nuclear accumulation (Fig. 4A–C). HeLa cells expressing STAT3 variants were in addition stained with an antibody against the phosphorylated tyrosine 705 epitope for the additional visualization of tyrosine-phosphorylated STAT3. This was followed by staining with a Cy3-labelled (red) secondary antibody for cells expressing STAT3-GFP, while cells expressing STAT3-SNAP constructs were stained using a Cy2-labelled (green) secondary antibody. Staining of cells with a phosphotyrosine-specific STAT3 antibody confirmed the translocation of the activated proteins upon IFNγ stimulation (Fig. 4D).

### STAT3-D427H shows increased DNA-binding activity and unaltered transcriptional response

**Fig. 5:**
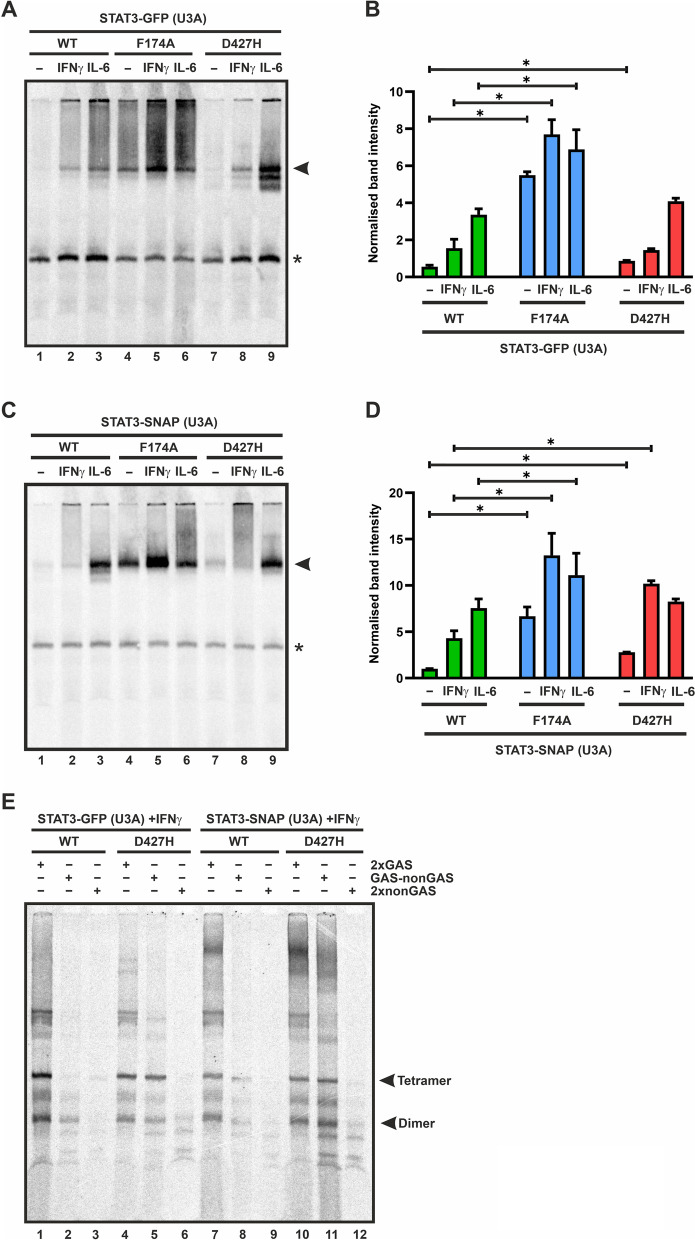
**A**–**E**: Increased DNA affinity of the D427H mutant. Electrophoretic mobility shift assays (EMSA) were performed using lysates from IL-6- and IFNγ-stimulated U3A cells expressing STAT3-GFP/-SNAP variants incubated with a [^33^P]-radioactively labelled DNA probe containing a consensus GAS sequence. Autoradiograms displaying the increased DNA-binding of the D427H-GFP mutant (**A**, **B**) and D427H-SNAP mutant (**C**, **D**) upon cytokine stimulation to a single GAS element (M67) as compared to the WT protein and similar to the positive control F174A mutant, including the quantification thereof from three independent transfection experiments. **E** Gelshift demonstrating the reduction in sequence-specificity for the STAT3-D427H mutant. Whole cell extracts from IFNγ-stimulated U3A cells transfected with GFP- or SNAP-tagged STAT3 variants were incubated with radioactively labelled DNA probes comprising of two complete GAS sites in tandem orientation (2xGAS), one-and-a half GAS site (GAS-nonGAS), or none GAS elements (2xnonGAS). DNA-bound STAT3 was separated by electrophoresis and visualized in the autoradiogram. Means ± standard deviations from three independent experiments with *p ≤ 0.05. Arrows indicate the respective STAT3-GFP or STAT3-SNAP bands of interest

Due to its spatial proximity to DNA in the crystal structure of DNA-bound STAT3 (Fig. 1B), we investigated the DNA-binding activity of the D427H mutant by means of electrophoretic mobility shift assays. Total cell extracts from untreated or cytokine-treated U3A cells expressing the STAT3 variants were incubated for 15 min with a [^33^P]-radioactively labelled M67 probe comprising a single high-affinity GAS binding site. The generated DNA-bound STAT3 complexes were then separated by electrophoresis and detected by autoradiography. Upon cytokine stimulation, the STAT3-D427H mutant showed increased DNA-binding ability compared to the WT protein (Fig. 5A-D). An enhanced DNA-binding activity was also reported for the homologous mutant E421K in STAT1 [17].

We then tested the specificity of the D427H mutant for binding to high-affinity STAT-specific sites on DNA. This was done by incubating total cell extracts from IFNγ -stimulated cells expressing STAT3 variants with DNA probes having either two complete GAS sites in tandem orientation (2xGAS), or one complete and one partial GAS element (GAS-nonGAS), or no GAS site (2xnonGAS). As described above, the STAT3-D427H showed an increased tetrameric and dimeric binding to GAS-nonGAS sequences as compared to the STAT3-WT, which bound weakly and predominantly as a dimer (Fig. 5E, lanes 2 and 5, and lanes 8 and 11). There was even a weak binding of the D427H mutant to 2xnonGAS (Fig. 5E, lanes 6 and 12).

**Fig. 6:**
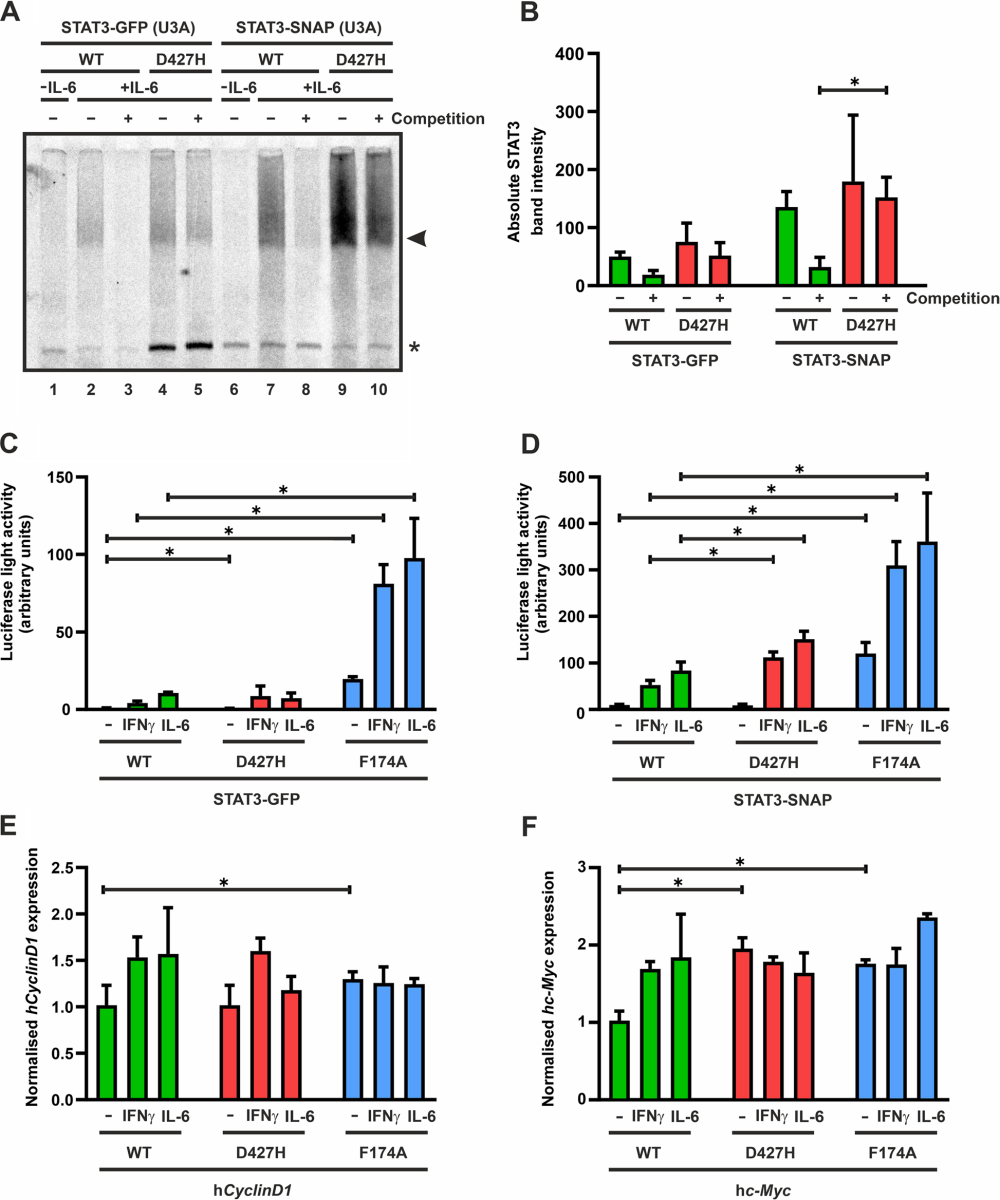
**A**–**F**: The D427H mutation in STAT3 markedly reduces its dissociation from DNA but does not grossly alter transcriptional activity. **A**, **B** Electrophoretic mobility shift assay displayed the reduced dissociation of D427H complexes from radioactively labelled M67 GAS sites on DNA upon competition with a 750-fold molar excess of unlabelled M67 GAS probes. Extracts from GFP- and SNAP-STAT3 expressing IL-6-stimulated U3A cells were incubated with [^33^P]-radioactively labelled M67 probe for 15 min before adding an excess of unlabelled GAS probes for 10 min. Autoradiogram depicts the increased residence time of STAT3-D427H on DNA (**A**) and the quantification thereof from three independent transfection experiments (**B**). Arrows indicate the respective STAT3-GFP or STAT3-SNAP bands of interest. **C**, **D** Luciferase reporter gene assays in reconstituted U3A cells expressing the indicated GFP-tagged (**C**) or SNAP-tagged STAT3 variants (**D)** normalized to the expression level of constitutively co-expressed β-galactosidase. The reporter construct used in these experiments contained triple GAS sites of the *Ly6E* gene (3xLy6E) in its promoter. Cells were left untreated or stimulated for 6 h with 50 ng/ml of recombinant IFNγ or 25 ng/ml of IL-6 before luciferase luminescence and the enzymatic activity of the co-expressed β-galactosidase were measured in whole cell extracts. The experiment was repeated in six independent transfections at least three times. **E**, **F** Endogenous gene expression by the STAT3 mutants was determined by real-time PCR. Data depict expression levels of the *CyclinD1* (**E**) and *c-Myc* (**F**) genes before and after 3 h stimulation with IL-6 or IFNγ. Gene induction was normalized to the expression of the house-keeping gene *GAPDH*. Histograms show means and standard deviations, wherein significant differences for comparison to the WT protein are marked by asterisks. The experiment was repeated three times in two independent transfections

To further investigate whether the D427H mutation has any impact on the dissociation from GAS sites on DNA, we performed competition experiments by challenging pre-formed STAT3 complexes on radioactively labelled M67 GAS probes with a 750-fold molar excess of unlabeled, double-stranded M67 oligonucleotides. As can be seen in Fig. 6A and B, the GAS-bound D427H mutant exhibited a significantly reduced dissociation from DNA and resisted competition upon the addition of excess unlabelled oligonucleotides, as compared to the WT protein which displayed a complete loss of radioactively labelled GAS-bound STAT3 in competition reactions. This indicated that the interchange of negatively charged aspartic acid residue with positively charged histidine at position 427 increased the affinity of STAT3 to GAS sites on DNA while concomitantly reducing its specificity and dissociation from DNA.

Based on the impact of the D427H mutation on DNA-binding of STAT3, we next studied whether the enhanced DNA-binding ability of STAT3-D427H corresponds to an increased transcriptional activity. For this purpose, we performed reporter gene assays in U3A cells by means of the synthetic luciferase reporter construct 3xLy6E which contains three GAS binding sites in its promoter region [20]. Since in these gene expression experiments endogenous STAT3 can possibly interfere with results from the over-expressed recombinant GFP- or SNAP-tagged variants, we firstly quantified the percentage of endogenous STAT3 expression in relation to the total cellular STAT3 expression using Western blot experiments. Compared to the levels of the transfected recombinant GFP- or SNAP-tagged STAT3 variants, the endogenous STAT3 expression was very low (14.7 ± 3.9% in IL-6-stimulated and 11.5 ± 3.4% in IFNγ-stimulated U3A cells). In transfected cells co-expressing recombinant SNAP-tagged STAT3, there was a similarly low percentage of native STAT3 as compared to total intracellular STAT3 pool (7.8 ± 1.2% in IL-6- and 11.5 ± 3.4% in IFNγ-treated cells).

U3A cells were transfected with plasmids coding for the STAT3 variants, the luciferase reporter construct and a plasmid coding for β-galactosidase used as a transfection control. On the next day, the cells were stimulated with IL-6 or IFNγ for 6 h. As demonstrated in Fig. 6C, D, and Supplementary data 1, both GFP- and SNAP-tagged D427H mutants showed no or only a mild change in reporter activity as compared to the corresponding WT protein, while the positive control F174A mutant showed the highest reporter activity, as is consistent with previous studies [18].

We also tested the induction of STAT3 target genes by the D427H mutant through real-time PCR (qPCR) experiments. U3A cells transfected with the STAT3 variants were serum-starved for 18 h prior to stimulation with IL-6 or IFNγ for 3 h. RNA was extracted from stimulated cells and converted to cDNA for qPCR experiments. The D427H mutant did not show an induction of the *CyclinD1* and *c-Myc* genes which surpassed the levels induced by the WT protein or the F174A mutant (Fig. 6E, F, Supplementary data 2). Surprisingly, the increased DNA-binding seen in the D427H mutant did not translate into a higher transcriptional activation as compared to the WT protein. This was also reported in the behaviour of the homologous STAT1-E421K mutation, wherein a higher residence time on DNA did not lead to higher induction of target genes [17].

## Discussion

In this study, we characterized the D427H mutation in the DNA-binding domain of STAT3 that was previously identified in patients with mature T-cell lymphomas, wherein it displayed hyper-phosphorylation and increased transcriptional response [16]. In an attempt to investigate the molecular mechanisms underlying this hyper-activity from an exchange of aspartic acid at position 427 with histidine, we introduced this mutation in plasmids encoding STAT3 with a carboxy-terminal GFP- or SNAP-fusion tag. Our results demonstrate that STAT3-D427H showed unaltered cytokine-induced tyrosine phosphorylation and nuclear accumulation but displayed an increased DNA-binding ability on STAT-specific sites as compared to the WT molecule. Furthermore, the D427H mutation reduced the specificity of STAT3 to high-affinity GAS sites and the dissociation rate of STAT3-DNA complexes. As a result, this enhanced binding to DNA in STAT3-D427H did not reflect as a corresponding increase in the inducibility of luciferase reporter activity and STAT3 target genes, which did not exceed beyond the levels induced by the WT protein.

While the position of the D427 residue could not be defined in the crystal structure of STAT3 due to the inherent structural flexibility of this region in the DNA-binding domain, the homologous residue E421 in the STAT1 molecule has been reported to be adjacent to the DNA molecule with its side chain pointing to the backbone of the DNA double helix [17]. The STAT1-E421K mutant displayed hyper-phosphorylation, prolonged nuclear accumulation and enhanced DNA-binding activity as well as a significantly reduced dissociation rate from DNA and unaltered transcriptional activation. This phenomenon was explained by the exchange of the negatively charged glutamic acid residue with positively charged lysine which increased the binding affinity of the mutant to the negatively charged DNA double helix, independent of the sequence specificity.

Similarly, the enhanced DNA binding seen in STAT3-D427H can be explained by the increased affinity conferred by the positively charged histidine compared to the negatively charged, native aspartic acid residue. However, this amino acid exchange at position 427 neither altered the cytokine-induced tyrosine phosphorylation nor the nuclear accumulation of STAT3-D427H in our experiments, underscoring the different behaviour of STAT3 and STAT1 around homologous mutations. While enhanced DNA-binding activity was seen for both STAT3-D427H and STAT1-E421K mutants, we also observed a similar reduced dissociation rate of the mutant STAT3-DNA complex, indicating that these two homologous mutations display similar but not identical phenotypes. Furthermore, in an alternative crystal structure of an unphosphorylated STAT3 parallel dimer lacking reciprocal phosphotyrosine-SH2 interactions, the D427 residue does not appear to contribute to the stability of this structure [22].

Interestingly, the enhanced affinity of STAT3-D427H to DNA did not reflect as an increase in its transcriptional activation. This phenomenon indicates that STAT-dependent gene transcription does not solely depend on the affinity of STAT proteins to GAS elements but suggests the presence of an additional mechanism that confers specificity to the STAT-DNA interaction. The STAT3-D427H mutant strongly binds to DNA not just on specific sites but also to non-specific sequences, which are usually present outside of promoter regions. Thus, the increased DNA binding displayed by the mutant is counterbalanced by unspecific binding to non-GAS sequences, resulting in grossly unchanged transcriptional activation of endogenous target genes. An example of such a mechanism has been discovered for STAT1, wherein residues E559 and E563 in the STAT1 linker domain regulate the dissociation of STAT1 from low-affinity sites and the residue K567 confers GAS recognition to STAT1 [23]. However, whether a similar mechanism exists in the STAT3 protein is currently unknown.

Activating mutations reported in the STAT3 protein usually alter one or more of its downstream signalling activities and/or its interactions with other proteins and components of the JAK-STAT pathway. In our experiments with STAT3-D427H, except for an increased binding affinity to DNA, all other signal transduction processes were unaffected. Therefore, we hypothesize that the residue D427 partially regulates the strength of the STAT3-DNA complex at transcriptionally inert sites. This aspartic acid residue possibly helps to select high-affinity GAS sites that foster the formation of a STAT3-DNA complex through additional interactions with the neighbouring residues that contribute to GAS recognition. This mechanism may ensure that GAS-like or non-GAS sequences are excluded from the formation of a strong STAT3-DNA complex due to the lack of sequence recognition. The negatively charged aspartic acid at position 427 plays a role in the release of STAT3 from the negatively charged DNA backbone.

In our experiments, we used U3A cells lacking endogenous STAT1 expression to eliminate any interference from STAT1/STAT3 heterodimers which are inevitably formed in cells with preserved STAT1 expression. We chose the STAT1-negative U3A cell line to eliminate any potential effect of STAT1 cross-interactions on the intracellular behavior of our recombinant STAT3 variants. Although the expression level of endogenous STAT3 in these cells is low compared to the transfected GFP- or SNAP-tagged variants, the fact that a residual expression of native STAT3 is nonetheless detectable must be considered an important limitation of our study.

In all our experiments, the behaviour of STAT3-D427H was compared to the WT protein and a previously described activating mutation, F174A, in the coiled-coil domain of STAT3. The F174 residue (F172 in STAT1) interacts with residues in the DNA-binding domain that stabilizes the anti-parallel dimer of STAT3 [18]. This mutation disrupts the anti-parallel binding interface of STAT3 such that it constitutively exists as a parallel dimer, thereby rendering it hyper-phosphorylated and selectively nuclear upon cytokine stimulation, resulting in an increased transcriptional response. Since the transcriptional activation by STAT3-D427H was relatively unchanged in comparison to the WT protein, it is unlikely that this residue, that does not have a precise role in the dimerization or nuclear localization of STAT3 but only a moderate influence on the DNA-binding affinity, bears a significantly increased oncogenic potential.

In summary, it is evident from our data that the D427H mutation increases the DNA-binding affinity of STAT3 but lowers sequence specificity. Although the STAT3-D427H mutation has been reported in the pathogenesis of NK/T-cell lymphoma, it is unlikely that the sole presence of this mutation could significantly increase the oncogenic potential of the STAT3 molecule. We propose that the STAT3-D427H mutation has, if any, only a mild impact on the etiology of NK/T-cell lymphomas. Furthermore, our data support previous studies suggesting that an increased affinity to DNA does not directly translate into an increased target gene induction by STAT proteins. These transcription factors have additional mechanisms conferring sequence recognition which help discriminate against inert sites to ensure optimal gene induction in response to cytokine signalling.

## Conclusion

Using site-directed mutagenesis, we investigated the phenotype of the point mutation D427H in the DNA-binding domain of STAT3, which was previously reported to exhibit GOF behaviour in NK/T-cell lymphoma patients. Our study did not reveal a hyper-activation of this mutant when compared with the GOF mutant F174A in the coiled-coil domain of STAT3. The D427H mutation displayed unaltered cytokine-induced tyrosine phosphorylation, nuclear accumulation, and subsequent transcriptional activation of STAT3. Similar to its homologous mutation in STAT1, the STAT3-D427H showed a reduced specificity to STAT-specific, high-affinity GAS sites on DNA coupled with a compromised dissociation rate from these sites, indicating that the mutation did not harbour the potential to grossly affect signal transduction by STAT3. Based on these findings, we conclude that STAT3-D427H may not have an oncogenic potential that exceeds the WT protein and may have little significance or an ancillary role in the pathogenesis of NK/T-cell lymphomas.

## Supplementary Information

**Additional file 1: Supplementary data 1.** Raw data from the representative reporter gene assays performed on U3A cells transfected with the indicated STAT3-GFP or STAT3-SNAP variants, normalized to their respective co-transfected b-galactosidase activity.

**Additional file 2: Supplementary data 2.** Raw data from the representative qPCR experiments performed on U3A cells transfected with the indicated STAT3-GFP variants, normalized to the expression of the house-keeping gene *GAPDH*.

**Additional file 3. **Raw data from Western blots and gelshifts.

## Data Availability

The raw data used in the present study would be available from the corresponding author on reasonable request.

## Abbreviations

APRF: Acute phase response factor
DMEM: Dulbecco’s modified Eagle’s medium
DTT: Dithiothreitol
EDTA: Ethylenediaminetetraacetic acid
EGTA: Ethylene glycol-bis(β-aminoethyl ether)-N,N,N′,N′-tetraacetic acid
EMSA: Electrophoretic mobility shift assay
FATHMM: Functional analysis through hidden Markov models
FCS: Fetal calf serum
GAPDH: Glyceraldehyde-3-phosphate dehydrogenase
GAS: Gamma-activated site
GFP: Green fluorescent protein
GOF: Gain-of-function
HEPES: 4-(2-Hydroxyethyl)-1-piperazineethanesulfonic acid
IFN: Interferon
IL: Interleukin
JAK: Janus kinase
NKTL: NK/T-cell lymphoma
PAGE: Polyacrylamide gel electrophoresis
PBS: Phosphate-buffered saline
PTCL: Peripheral T-cell lymphoma
RPMI: Roswell Park Memorial Institute
SDS: Sodium dodecyl sulphate
PVDF: Polyvinylidene diflouride
RT: Room temperature
SIE: Sis-inducible element
qPCR: Real-time PCR
SH2: Src-homology 2
STAT: Signal transducer and activator of transcription
T-LGL: T-cell large granular lymphocytic leukemia
WT: Wild-type
