# Supplementary material for: Evaluation of the putative lymphoma-associated point mutation D427H in the STAT3 transcription factor
Source: BMC Mol Cell Biol. 2022 Jun 25;23:23. doi: 10.1186/s12860-022-00422-9 (PMC9233852; doi:10.1186/s12860-022-00422-9)

Figure 2 A

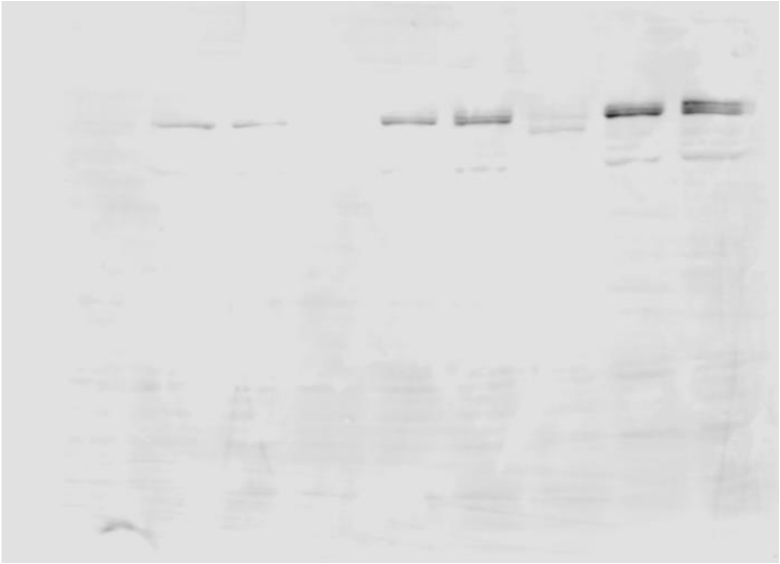

← P-STAT3-GFP IL-6 (U3A)

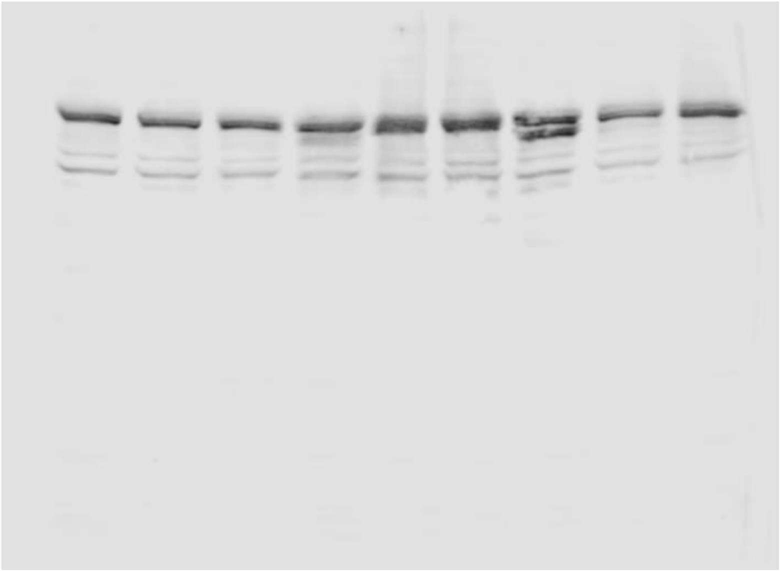

← STAT3-GFP IL-6 (U3A)

Figure 2 C

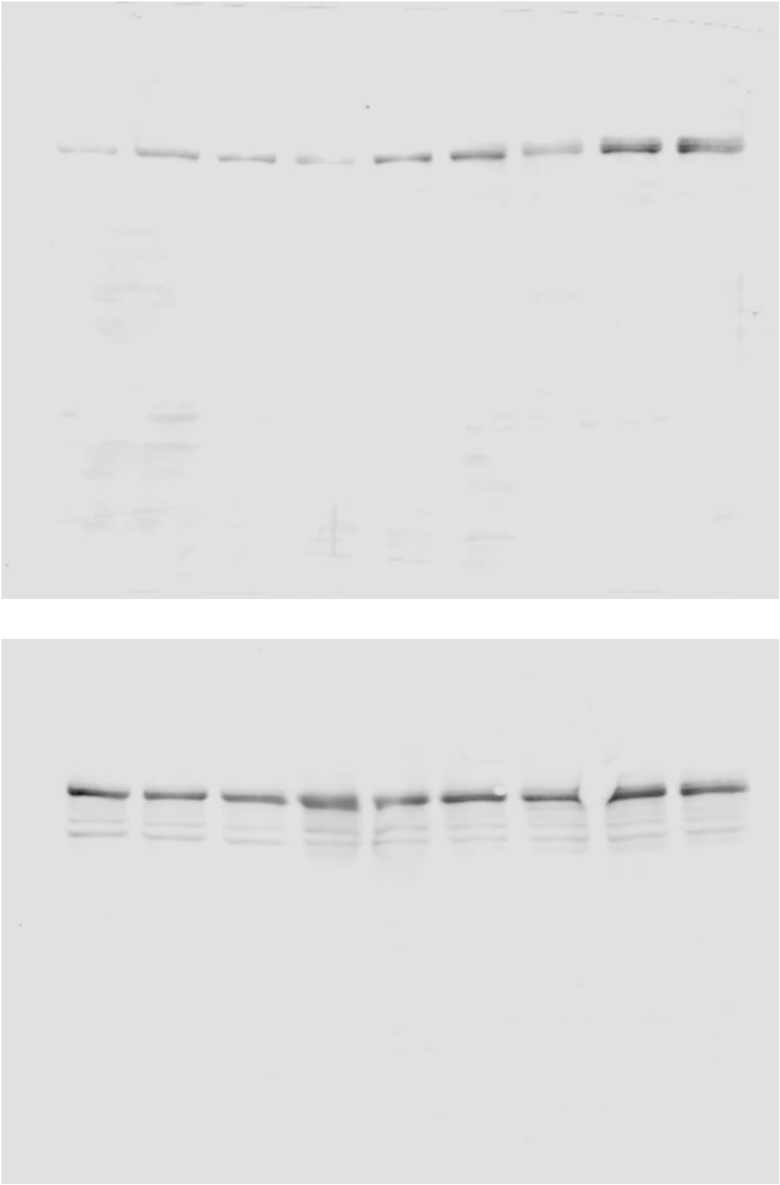

Figure 2 E

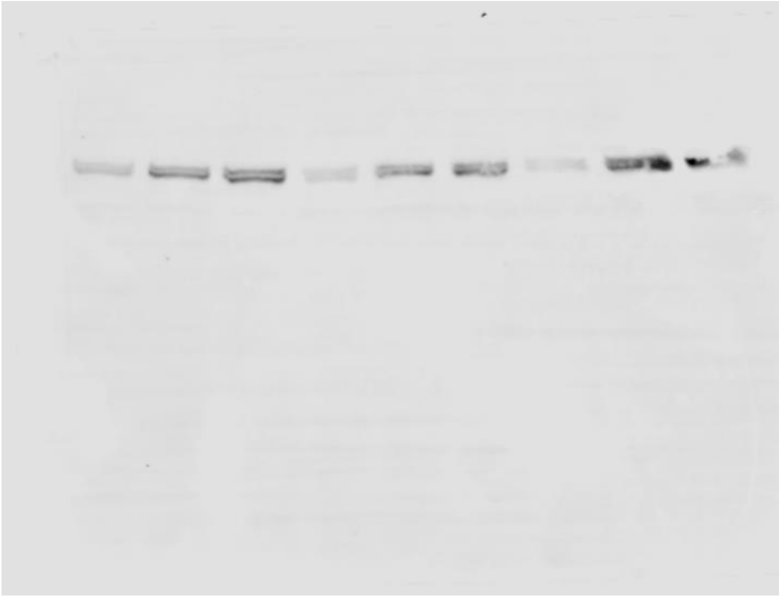

← P-STAT3-SNAP IL-6 (U3A)

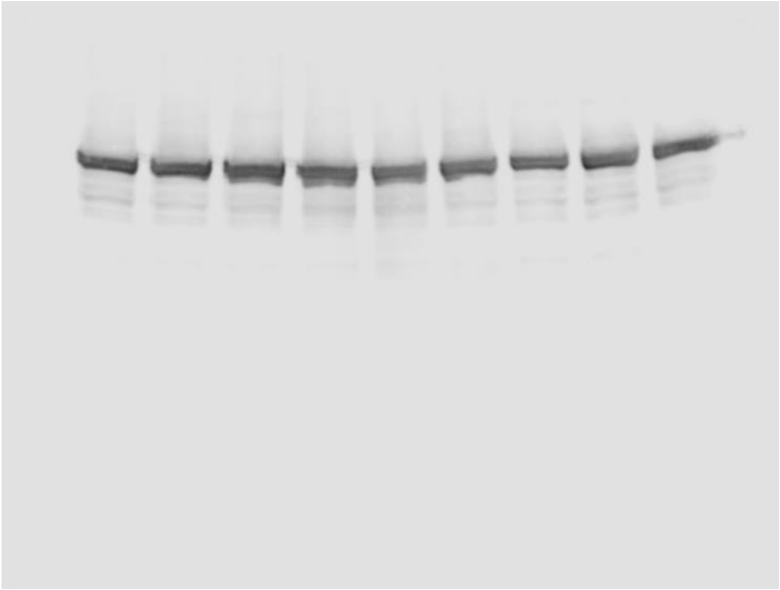

← STAT3-GFP IL-6 (U3A)

Figure 2 G

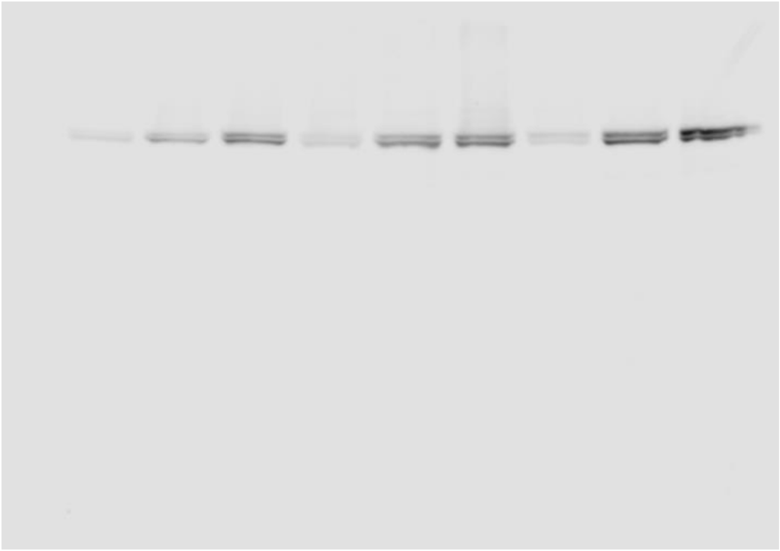

← P-STAT3-SNAP IFNγ (U3A)

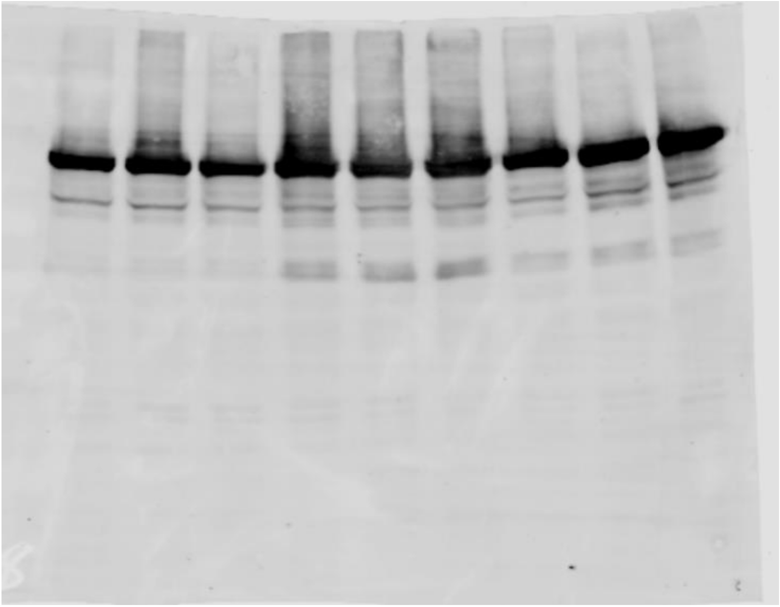

← STAT3-SNAP IFNγ (U3A)

Figure 2 I

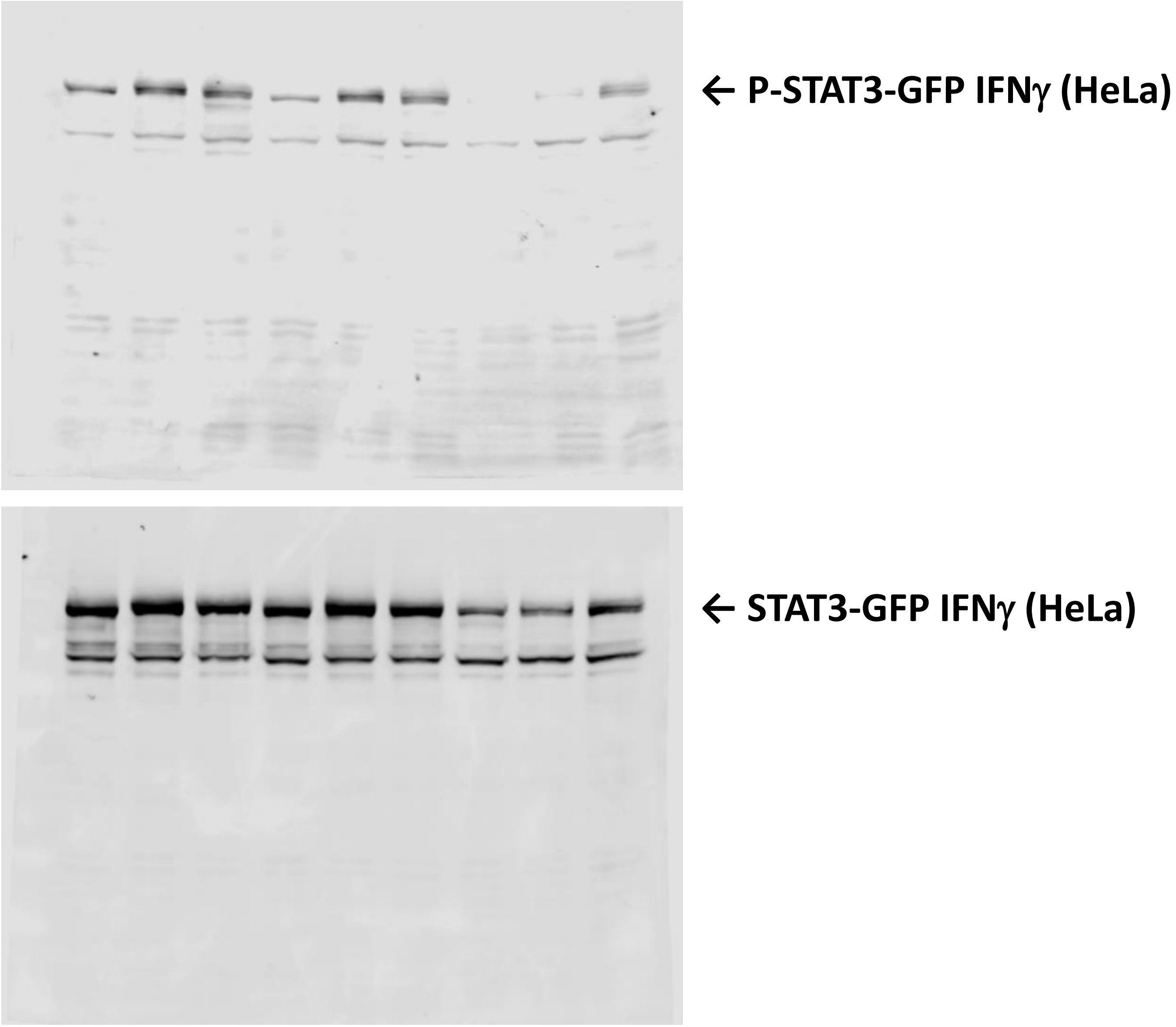

Figure 2 K

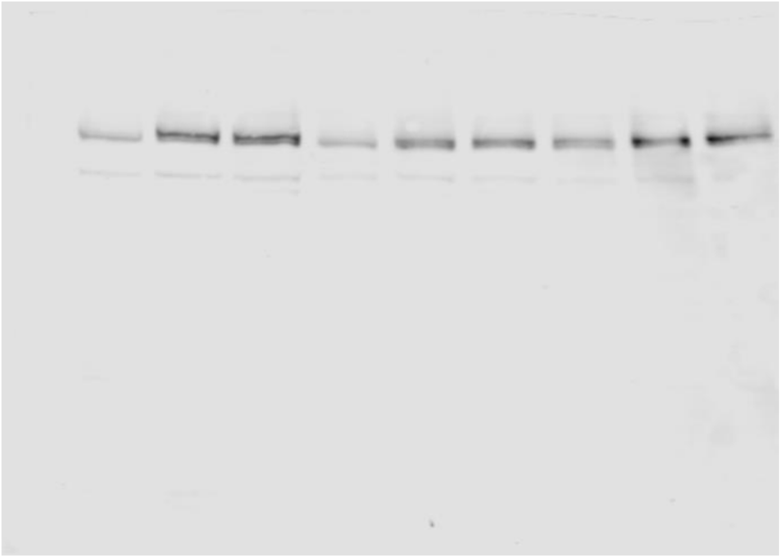

← P-STAT3-SNAP IFNγ (HeLa)

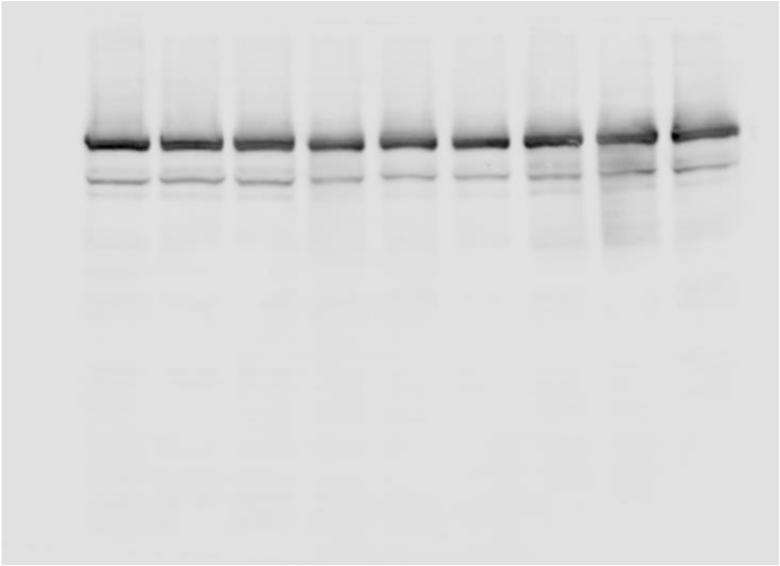

← STAT3-SNAP IFNγ (HeLa)

Figure 5 A

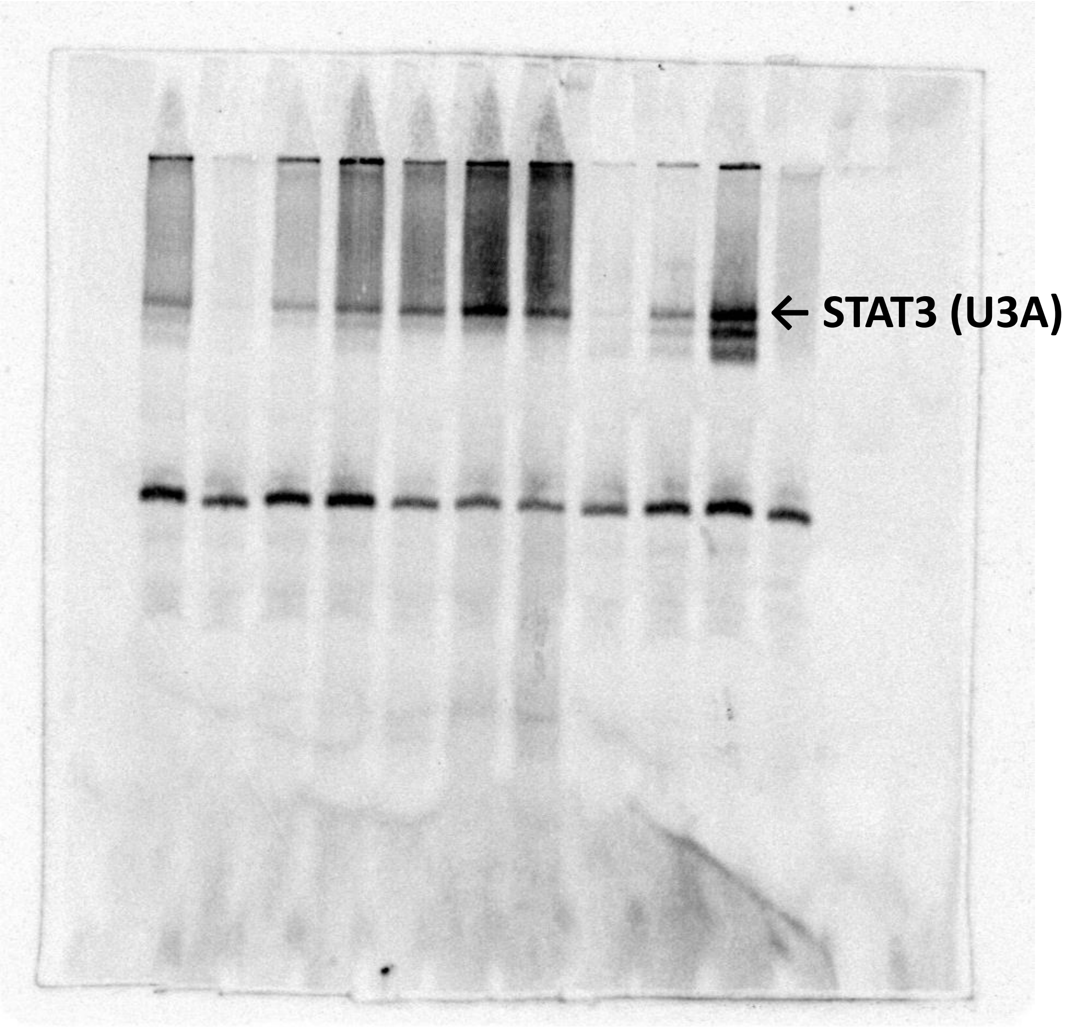

Figure 5 C

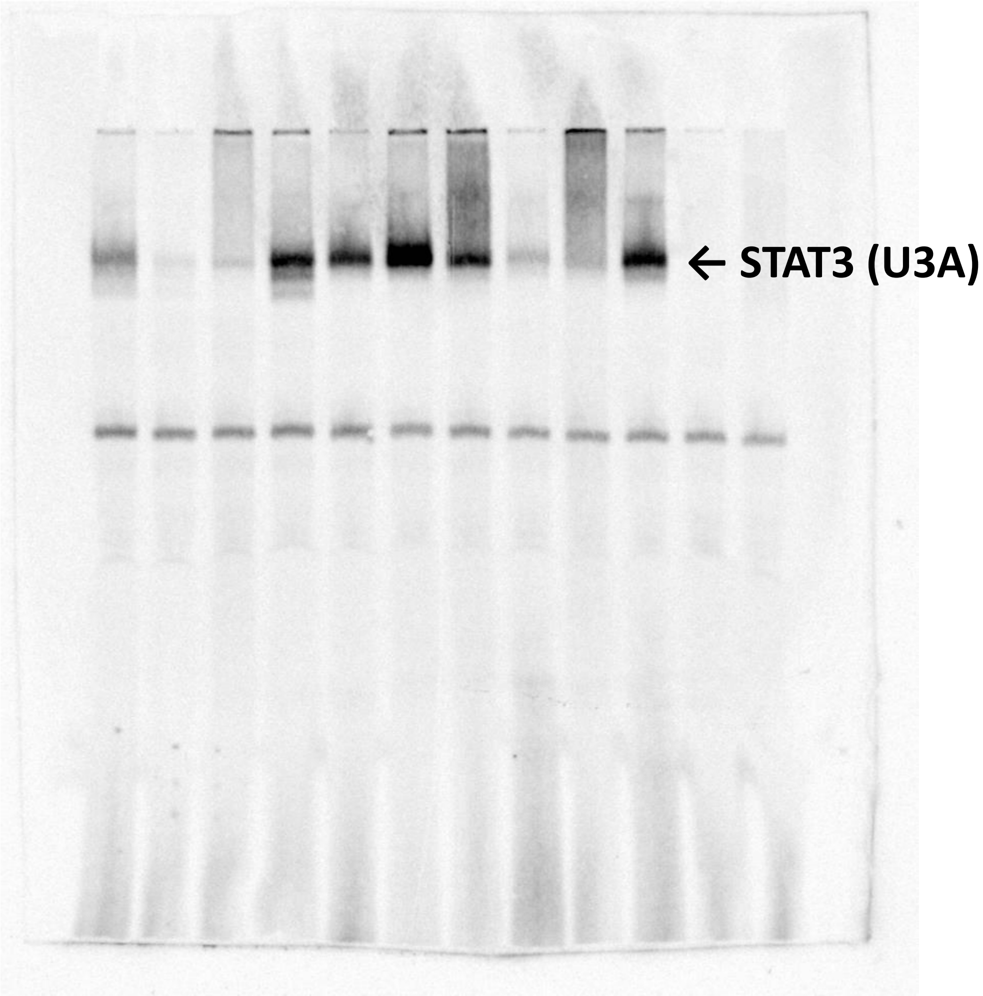

Figure 5 E

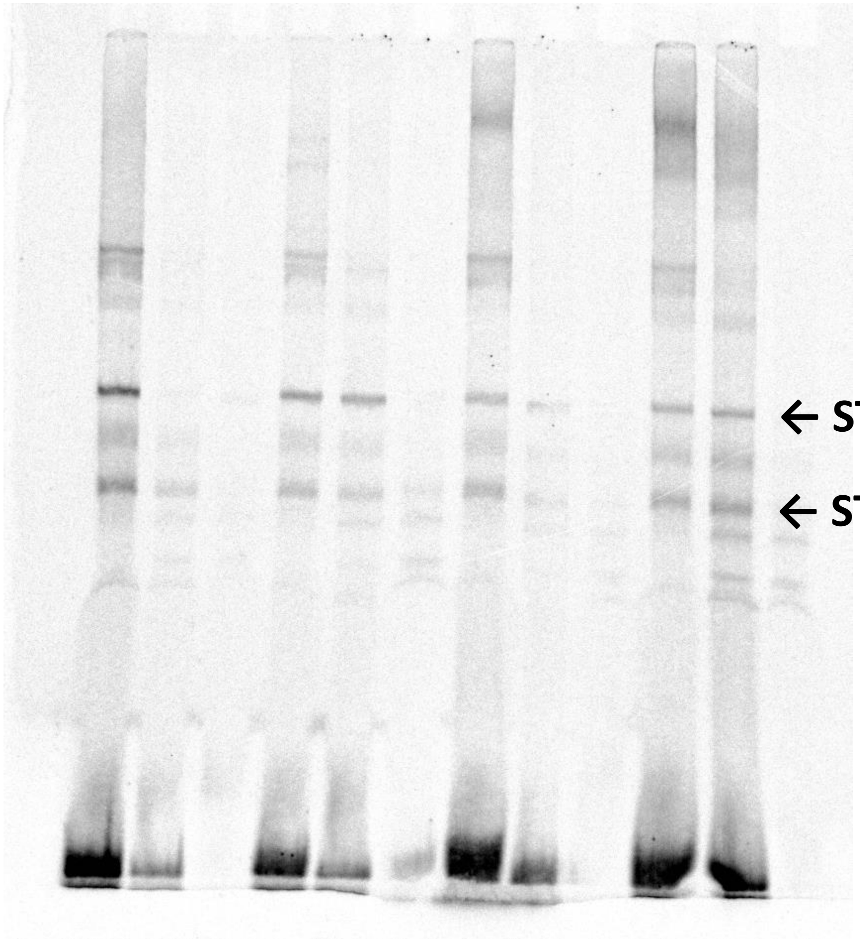

← **STAT3 (U3A) tetramer**

← **STAT3 (U3A) dimer**

Figure 6 A

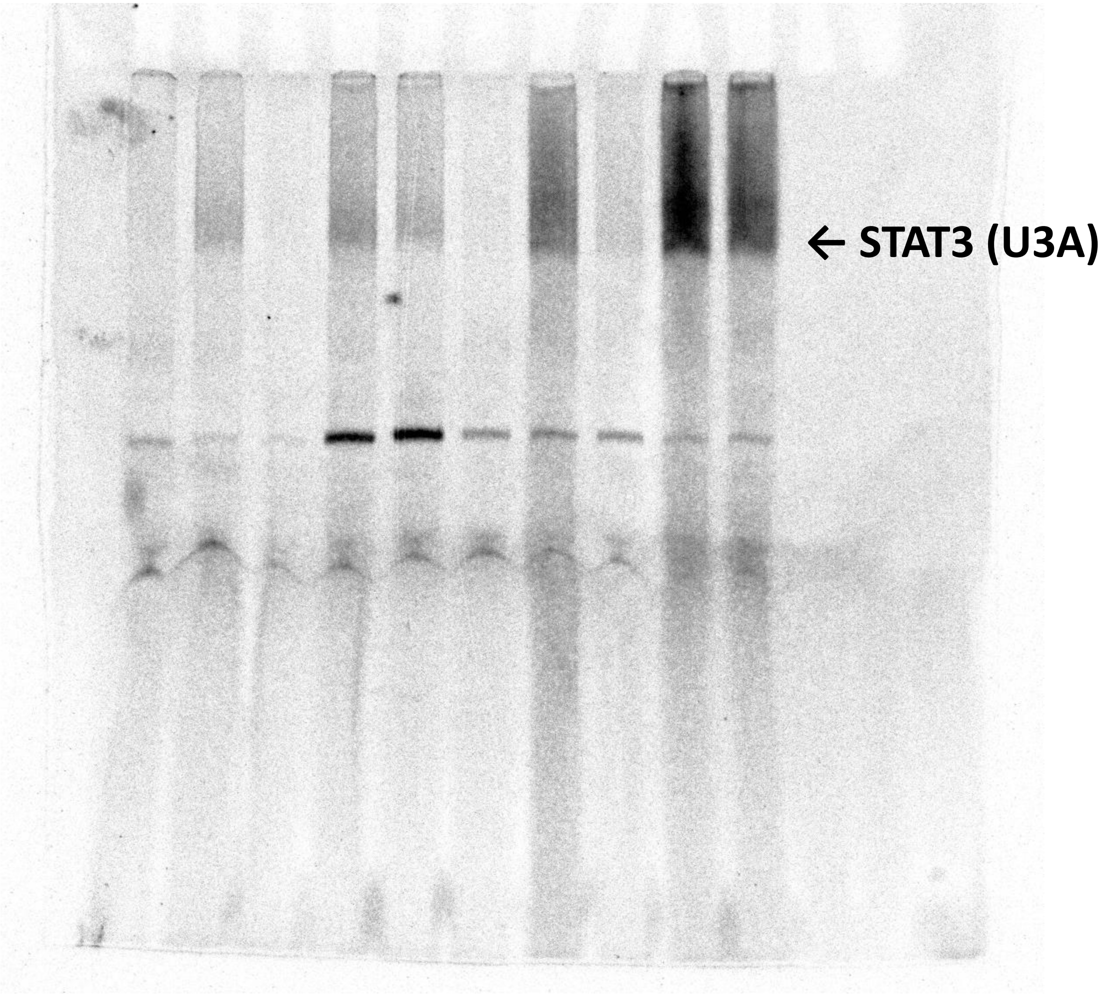

Supplement: Supplementary file 3 — Additional file 3. Raw data from Western blots and gelshifts. [file 12860_2022_422_MOESM3_ESM.pdf]
